# Clinical Manifestations and Molecular Backgrounds of Parkinson's Disease Regarding Genes Identified From Familial and Population Studies

**DOI:** 10.3389/fneur.2022.764917

**Published:** 2022-06-02

**Authors:** Kenya Nishioka, Yuzuru Imai, Hiroyo Yoshino, Yuanzhe Li, Manabu Funayama, Nobutaka Hattori

**Affiliations:** ^1^Department of Neurology, Juntendo University School of Medicine, Tokyo, Japan; ^2^Department of Research for Parkinson's Disease, Juntendo University Graduate School of Medicine, Tokyo, Japan; ^3^Research Institute for Diseases of Old Age, Graduate School of Medicine, Juntendo University, Tokyo, Japan

**Keywords:** familial Parkinson's disease, genetics, GWAS, dopamine, alpha-synuclein, LRRK2

## Abstract

Over the past 20 years, numerous robust analyses have identified over 20 genes related to familial Parkinson's disease (PD), thereby uncovering its molecular underpinnings and giving rise to more sophisticated approaches to investigate its pathogenesis. α-Synuclein is a major component of Lewy bodies (LBs) and behaves in a prion-like manner. The discovery of α-Synuclein enables an in-depth understanding of the pathology behind the generation of LBs and dopaminergic neuronal loss. Understanding the pathophysiological roles of genes identified from PD families is uncovering the molecular mechanisms, such as defects in dopamine biosynthesis and metabolism, excessive oxidative stress, dysfunction of mitochondrial maintenance, and abnormalities in the autophagy–lysosome pathway, involved in PD pathogenesis. This review summarizes the current knowledge on familial PD genes detected by both single-gene analyses obeying the Mendelian inheritance and meta-analyses of genome-wide association studies (GWAS) from genome libraries of PD. Studying the functional role of these genes might potentially elucidate the pathological mechanisms underlying familial PD and sporadic PD and stimulate future investigations to decipher the common pathways between the diseases.

## Introduction

The nature of Parkinson's disease (PD) was initially described by James Parkinson in his “Essay on the shaking palsy” in 1817. Since then, efforts have been made to understand the clinical symptoms and pathophysiology of this disease. However, currently, only incomplete symptomatic treatments are available. The common symptoms of PD are tremor, rigidity, akinesia, and unsteadiness. Age is an important prognostic factor that increases the prevalence of PD, with 41 patients in their 40s, 107 patients in their 50s, 428 patients in their 60s, 1,087 patients in their 70s, and 1,903 patients older than 80 years being detected (all per 100,000) (1, 2). PD is pathologically characterized by the degeneration of dopamine neurons in the substantia nigra and the deposition of Lewy bodies (LBs) or Lewy neurites, a pathological hallmark of PD, which are often observed in the affected regions (3). The major component of LBs is α-synuclein, encoded by the *SNCA* gene located in 4q21-22 (4). α-Synuclein is thought to be the key protein involved in the pathological mechanisms underlying PD and other neurodegenerative disorders.

The development of molecular genetics technologies and family tree analysis for PD have identified genes linked to PD (5–9). Over 20 genes, namely *PARK* genes from *PARK1* to *PARK23* from Online Mendelian Inheritance in Man (OMIM) (https://www.omim.org), are associated with the development of PD. However, the *PARK* genes include heterogeneous genes such as Mendelian genes, candidate loci, or genes not confirmed to mediate the disease pathogenicity (10). The *PARK* genes also include genes confirmed as genes not associated with typical PD (i.e., ATP13A2, associated with atypical parkinsonism) (11). *SNCA* and *LRRK2* have been identified using positional cloning in families with PD (7, 12–14) and were also later detected as major risk factors for PD using genome-wide association studies (GWAS) (15–18). The autosomal recessive genes inherited in families, *PRKN* (6) or *PINK1* (9), were not identified through the GWAS as common genetic risk variants probably due to their low prevalence. There are several large studies that reported a lack of association between heterozygous PRKN and PINK1 variants with PD (19–21), while PD risk might be increased with heterozygous variants in these genes (22).

This review aimed to describe the clinical differences among patients with various pathogenic genes associated with PD or Parkinsonism to highlight potential underlying mechanisms regulating these genes, with a particular focus on *SNCA, LRRK2, VPS13C, glucosylceramidase beta* (*GBA1*), *GCH1*, and *microtubule-associated protein tau* (*MAPT*). These genes have been identified as PD causative or susceptible genes in PD families and were found through meta-analyses of GWAS (15–18). We aimed to identify the common pathological pathways governed by these genes between familial and sporadic PD.

## *PARK* Genes

Genes associated with familial PD were historically categorized as *PARK*. To date, the genes belonging to the PARK category range from *PARK1* to *PARK24* (Table 1) (OMIM: https://www.ncbi.nlm.nih.gov/omim), with *PARK1* being the same as *PARK4*. The *PARK* category includes twelve autosomal dominant inheritances, nine autosomal recessive inheritances, one X-linked, and four unidentified genes. Although the *PARK16* locus (1q32) is a prominent risk locus associated with PD, responsible genes have not been determined (15). Other genes excluded from the *PARK* category, such as *GBA1, GTP cyclohydrolase 1* (*GCH1*), and *MAPT*, were also significantly linked to PD or parkinsonism through meta-analyses of GWAS (17, 18, 23).

**Table 1:** *PARK* categories from the genes related to PD.

**Locus (OMIM #)**	**Location**	**HUGO gene name**	**Gene symbol**	**Disease onset**	**Inheritance**	**LB pathology**	**Genes appeared by GWAS**
PARK1 (163890)	4q22.1	Synuclein alpha	*SNCA*	Young- or middle-aged onset	AD	+++	+
PARK2 (602544)	6q26	Parkin RBR E3 ubiquitin-protein ligase	*PRKN*	Young- or juvenile-onset	AR	–	
PARK3 (NA)	2p13		*PARK3*	Late-onset	AD		
PARK4 (163890) = PARK1	4q22.1	Synuclein alpha	*SNCA*	Young- or middle-aged onset	AD	+++	+
PARK5 (191342)	4p13	Ubiquitin C-terminal hydrolase L1	*UCHL1*	Young- or middle-aged onset	AD		
PARK6 (608309)	1p36	PTEN induced kinase 1	*PINK1*	Young-onset	AR	–	
PARK7 (602533)	1p36.23	Parkinsonism associated deglycase	*PARK7*	Young-onset	AR		
PARK8 (609007)	12q12	Leucine-rich repeat kinase 2	*LRRK2*	Late-onset	AD	–, + or +++	+
PARK9 (610513)	1p36.13	ATPase cation transporting 13A2	*ATP13A2*	Young-onset	AR	–	
PARK10 (NA)	1p32	Parkinson disease 10 (susceptibility)	*PARK10*	Late-onset	Unclear		
PARK11 (612003)	2q37.1	GRB10 interacting GYF protein 2	*GIGYF2*	Late-onset	AD		
PARK12 (NA)	Xq21-q25	Parkinson disease 12 (susceptibility)	*PARK12*	Late-onset	X-linked		
PARK13 (606441)	2p13.1	HtrA serine peptidase 2	*HTRA2*	Young- and late-onset	AD		
PARK14 (603604)	22q13.1	Phospholipase A2 group VI	*PLA2G6*	Young-onset	AR		
PARK15 (605648)	22q12.3	F-box protein 7	*FBXO7*	Young-onset	AR		
PARK16 (NA)	1q32	Parkinson disease 16 (susceptibility)	*PARK16*	Late-onset	Unclear		
PARK17 (601501)	16q11.2	VPS35 retromer complex component	*VPS35*	Late-onset	AD		
PARK18 (600495)	3q27.1	Eukaryotic translation initiation factor 4 gamma 1	*EIF4G1*	Late-onset	AD		
PARK19 (608375)	1p31.3	DnaJ heat shock protein family (Hsp40) member C6	*DNAJC6*	Young-onset	AR		
PARK20 (604297)	21q22.1	Synaptojanin 1	*SYNJ1*	Young-onset	AR		
PARK21 (614334)	20p13	DnaJ heat shock protein family (Hsp40) member C13	*DNAJC13*	Late-onset	AD		
PARK22 (616244)	7p11.2	Coiled-coil-helix-coiled-coil-helix domain containing 2	*CHCHD2*	Late-onset	AD	+++	
PARK23 (608879)	15q22.2	Vacuolar protein sorting 13 homolog C	*VPS13C*	Young-onset	AR	+++	+
PARK24 (176801)	10q22.1	Prosaposin	*PSAP*	Middle- or late-onset	AD		
**Non-categorized genes in PARK**							
(600225)	14q22.2	GTP cyclohydrolase 1	*GCH1*	Young-onset	AD	–	+
(606463)	1q22	Glucosylceramidase beta	*GBA1*	Young-onset	AR	+++	+
(NA)	5q34	ATPase phospholipid transporting 10B (putative)	*ATP10B*	Young-onset	AR		

*OMIM, Online Mendelian Inheritance in Man; HUGO, human genome organization; AD, autosomal dominant; AR, autosomal recessive; NA, not applicable*.

The prevalence of familial PD among all patients with PD is ~10–20% (24), whereas the rest of the cases without any family history are considered sporadic PD (80–90%). LRRK2 p.G2019S is the most common mutation in specific populations, such as in 30% cases of the Ashkenazi Jews or Arab Berbers. In other populations, the prevalence of *LRRK2* was estimated at 2–5% (25). There are very few other pathogenic genes involved in PD, showing a prevalence of 1–3% among familial PD (26–34). Overall, the prevalence of pathogenic genes is extremely low among both familial and sporadic PD.

## Genome-Wide Association Studies

Several meta-analyses of GWAS have been performed to identify the molecular mechanisms regulating PD (15–18, 23). Based on the analyses from over a million patients and controls, common genes associated with the PD cohort were *PARK16, GBA1, SNCA, LRRK2, GCH1*, and *VPS13C*, with *SNCA* and *LRRK2* showing a significantly higher association with PD than other genes across populations (15, 18). Moreover, another gene, *MAPT*, has been identified to be associated with the PD cohort. In the European cohort, *SNCA, GBA1*, and *LRRK2* are significantly associated with PD (17, 23). In the Asian cohort, *SC2C, WBSCR17*, and *BST1* showed a robust association with PD (15, 18). Intriguingly, the fact that familial PD genes have been identified by GWAS means that familial PD genes are involved in the pathogenesis of sporadic PD, strongly suggesting common pathogenic pathways between familial and sporadic PD, or that multiple concurrent variants of familial PD genes may relate to the rapid motor progression of sporadic PD (35).

In the next section, we have described the genetic evidence, clinical and pathological features, and molecular backgrounds in terms of PD-associated genes. The main clinical features are also summarized in Table 2.

**Table 2:** Major clinical features for each gene.

**Genes**	**Clinical features**
***SNCA***	Young- or middle-aged onset of parkinsonism, cognitive decline, psychosis, consciousness fluctuation, resembling the symptoms of PDD or DLBs.
***LRRK2***	Middle- or late-onset of parkinsonism with an excellent response to levodopa, resembling the symptoms of sporadic PD.
***VPS13C***	Early- or middle-age onset with severe cognitive decline.
***GBA1***	Young-onset with cognitive decline, resembling the symptoms of DLBs. short survival times.
***GCH1***	Juvenile- or young-onset with dopa-responsive dystonia.

## Synuclein Alpha

### Clinical Symptoms of Patients With *SNCA* Variants

Synuclein alpha **(***SNCA*) variants associated with PD are of two types: one has missense mutations, such as p.A30G, p.A30P, p.E46K, p.H50Q, p.G51D, p.A53T/E/G/V, and p.E83Q, whereas the other has amplifications, including duplication and triplication (5, 8, 12, 36–43). Patients with missense variants are likely to develop parkinsonism in young- or middle-aged adults, along with cognitive decline or psychosis (26, 44–46). Patients with genetic amplifications showed young- or middle-aged onset of parkinsonism, psychosis, and consciousness fluctuation, resembling the symptoms of PD with dementia (PDD), along with LBs (47, 48). The amplified genes contain two- or three-fold tandem repeat replication of an *SNCA* locus (49). *SNCA* locus amplification induces an increased expression of α-synuclein in the brain or peripheral blood and accumulations of α-synuclein in the detergent-insoluble fraction (50). The clinical severity of patients with *SNCA* multiplications obeys the gene-dosage-dependent phenomenon (51). Patients with four copies of the gene show a more severe PD onset at a younger age (the 20–30s) than those with three copies (the 40–50s) (51). More copy numbers of *SNCA* may induce more severe symptoms, indicating that the increased intracellular concentration of α-synuclein is responsible for PD development.

The neuroimaging reports regarding familial PD are scarce. Most of the analyses were from the cross-sectional study without considering the duration between the disease onset and examination time. However, these differences may suggest that each variant has a different prognosis or a different spread of α-synucleinopathy. *SNCA* amplifications may present specific neuroimaging patterns related to dementia with LBs (DLBs) or PDD (26, 48). The brain magnetic resonance imaging (MRI) showed progressive atrophic changes in the hippocampus (26, 48), whereas [^123^I]N-ω-fluoropropyl-2β-carbomethoxy-3β-(4-iodophenyl) tropane (^123^I-FP-CIT) single-photon emission computed tomography (SPECT) showed a reduced expression of the dopamine transporter. [^123^I]metaiodobenzylguanidine (MIBG) myocardial scintigraphy showed a reduced heart-to-mediastinum ratio (52). The brain SPECT or positron emission tomography (PET) revealed hypoperfusion in the bilateral occipital lobes (48). Patients with a missense variant of *SCNA*, p.A53T, showed atrophic changes in the hippocampus and the temporal lobes in the brain MRI, a decreased heart-to-mediastinum ratio in MIBG myocardial scintigraphy, and hypoperfusion in the parieto-occipital lobe in the brain SPECT (53, 54). The findings infer that *SNCA* variants cause the widespread propagation of α-synuclein, with patients showing symptoms similar to DLB.

### Pathology of Patients With *SNCA* Variants

Patients with *SNCA* variants commonly show a severe neuronal loss in the substantia nigra or the hippocampus and widespread appearances of LBs and Lewy neurites (47, 55) with Braak's stage 5 or 6 (46, 54). Braak's staging is advocated to confirm the severity of LB formation (56) localized in the medulla oblongata in stage 1, the pontine tegmentum in stage 2, the midbrain in stage 3, the basal prosencephalon and mesocortex in stage 4, the neocortex in sensory association areas of the neocortex and prefrontal neocortex in stage 5, and the premotor and motor areas of the neocortex in stage 6. The higher stages include the pre-stage areas. The staging is based on the LB pathology that is widespread from the medulla oblongata to neocortices and depends on disease severity. Patients with *SNCA* variants commonly show the higher Braak's staging with DLB (57). Patients with *SNCA* triplication showed higher expression levels of α-synuclein in the blood and brain tissue (50). Moreover, disease onset correlates with *SNCA* gene dosage (51). The findings support the hypothesis that the expression levels of α-synuclein direct the clinical severity of PD in patients with *SNCA* multiplications.

### α-Synuclein and Lysosomal Storage Disorders

The abnormal expression and aggregation of α-synuclein are critical factors for PD, PDD, or DLB. α-synuclein-positive inclusions or LBs have been identified in several other disorders, such as multiple system atrophy (MSA) or pure autonomic failure, Alzheimer's disease, Down's syndrome, Hallervorden–Spatz disease, and Gaucher's disease (58–63). The physiological function and accumulation of α-synuclein are only partially understood. α-Synuclein is predominantly localized in presynaptic termini of neurons and regulates neurotransmitter release promoting sensitive factor attachment protein receptor (SNARE)-complex assembly (64, 65). α-Synuclein is subjected to lysosomal degradation by the autophagy–lysosomal systems (66) and the chaperon-mediated autophagy (67). Lysosomes play a central role in maintaining cellular metabolism, degradation, and recycling of amino acids and lipids, eliminating damaged proteins/organelles or proteins with pathogenic properties (66, 68). The lysosomes collaborate with micro-autophagy and macro-autophagy, chaperone-mediated autophagy, and endosomes to conduct their functions (67). Impaired lysosomal function induces the accumulation of aggregated α-synuclein and the formation of LB. Thus, lysosomal dysfunction induces dysfunctional protein and organelle accumulation, leading to lysosomal storage disorders. Several genes, such as *SNCA, LRRK2, GBA1, ATP13A2*, and *VPS35*, among the pathogenic ones related to familial PD, are associated with lysosomal storage disorders (68). Genetic screening for 54 genes related to lysosomal storage disorders has identified PD-related genes, such as *GBA1, SMPD1, CTSD, SLC17A5*, and *ASAH1* (69). Most patients with PD (56%), including 40% with familial and 60% with sporadic PD, have at least one putative damaging variant related to lysosomal storage disorders (69).

### Formation of LBs and Propagation of α-Synuclein Pathologies

It has been reported that a patient's brain having DLB shows a high accumulation of insoluble α-synuclein (70, 71). The membrane unbound form of α-synuclein is natively unfolded, whereas the elevated protein levels or pathogenic mutations of α-synuclein promote structural conversion to crossed β-sheets, leading to the accumulation of insoluble α-synuclein fibrils (72). Electron microscopy analysis reveals that the introduction of α-synuclein p.A53T mutation accelerates fibril formation with a twisted appearance (73). Other *SNCA* variants are also likely to facilitate the structural conversion and subsequent LB formation. The degrees of aggregation and fibril propagation by α-synuclein in the central nervous system probably determine the clinical severity of PD, PDD, or DLB obeying Braak's hypothesis rule (56). PD is now recognized as a systemic disease (74). The accumulation of α-synuclein aggregates is observed in the brain and the cardiac nerves, or Auerbach's or Meissner's plexus (75, 76). Concurrently, patients with PD show both motor symptoms and nonmotor symptoms (77). Motor symptoms include gait disturbance, tremor, and rigidity, whereas the nonmotor symptoms include persistent pain, insomnia, constipation, urinary incontinence, and orthostatic hypotension accompanied by syncope or faintness (77). The propagation and expansion of α-synuclein aggregates may be essential factors in determining the clinical severity and symptoms of PD.

### Propagation of α-Synuclein and Prion-Like Hypothesis

Animal models of α-synuclein propagation suggest that PD is a prion-like disease. Inoculation of α-synuclein derived from PD brain tissues with LBs replicates progressive nigral degeneration and triggers the pathological conversion of endogenous α-synuclein in mouse and monkey models (78). The inoculation of insoluble α-synuclein from the DLB brains also causes hyperphosphorylated α-synuclein pathology in mice (79). The inoculation of α-synuclein fibrils in mice expressing pathological human p.A53T mutant α-synuclein causes rapid propagation (80). These previous studies support the “prion-like hypothesis,” indicating how pathological α-synuclein derived from PD, DLB, or MSA, as well as fibrils prepared from recombinant protein, induces the cell-to-cell transmission, the spreading of α-synuclein, and amyloid-like formation.

## Genetic Evidence, Clinical and Pathological Features, and Molecular Backgrounds of Other Genes Associated With PD

### Glucosylceramidase Beta

The *GBA1* gene consists of 11 exons, 7.6 kb in length, and is located on chromosome 1q21 (81). *GBA1* pathogenic variants cause Gaucher disease (82, 83), a lysosomal storage disorder characterized by the deficiency of the enzyme glucocerebrosidase (GCase) (84). It is categorized into three types: type 1, non-neuropathic Gaucher disease with various types of symptoms and courses; type 2, acute neuropathic Gaucher disease with an infantile-onset and rapidly progressive neurological symptoms; and type 3, chronic neurological symptoms (84). Patients with type 2 and type 3 Gaucher disease commonly show neurological symptoms (84), such as parkinsonism, hydrocephalus, eye movement disorder, epilepsy, dementia, or ataxia. Pathologically, type 1 Gaucher disease presented numerous α-synuclein-positive inclusions similar to LBs in the hippocampus (60). Moreover, *GBA1* variants have a higher odds ratio, with approximately five-fold OD between PD vs. controls (85). Patients with *GBA1* pathogenic variants likely induce cognitive decline and short survival times, whose symptoms resemble DLBs with no or low levels of Alzheimer's disease (86–88). *GBA1* is involved in the glucolipid metabolism and hydrolyzes glucosylceramide to ceramide and glucose and glucosylsphingosine to sphingosine and glucose (84). It has been proposed that lysosomal impairment directly causes α-synuclein aggregation, leading to the pathogenesis of synucleinopathies (66, 89).

### *LRRK2* Gene

The pathogenic variants in the *LRRK2* gene are the most common genetic cause of familial PD (90). The prevalence of LRRK2 p.G2019S is over 30% in the Ashkenazi Jews or Arab Berber. Other populations essentially showed ~0–4% prevalence among sporadic and familial PD (25). *LRRK2* is located on 12q12, consists of 51 exons, and encodes a large protein with 2,527-amino acids that belong to the ROCO protein family and include seven domains: armadillo, ankyrin, leucine-rich repeat (LRR), Ras in complex proteins (Roc), C-terminal of Roc (COR), kinase, and WD40 (14). We originally mapped the region around 12p11.2–q13.1 from the Sagamihara family in Japan (7). Two reports concurrently identified the causative gene and mutations from Spanish, German–Canadian, and American families (13, 14). After numerous screening analyses, to date, seven missense mutations (p.N1437H, p.R1441C/G/H, p.Y1699C, p.G2019S, and p.I2020T) are thought to be pathogenic variants from the pathological observations (91).

Patients with *LRRK2* variants show middle- or late-onset parkinsonism with an excellent response to levodopa (25, 90). Their clinical course resembles that of sporadic PD. *LRRK2* showed broad types of brain pathologies, including LB pathology, tau pathology, TDP-43 pathology, or isolated nigral degeneration (91, 92). LRRK2 p.G2019S, the most prevalent variant, commonly showed LB pathology with broad severities of Braak's stage from 3 to 6 and rarely involves tau pathology (91). On the other hand, tau pathology is found in almost 100% of the p.G2019S carriers (93). A Japanese PD family with LRRK2 p.I2020T also showed a variety of pathological changes, including LB formation and glial cytoplasmic inclusion (94). Moreover, patients with LRRK2 p.R1441G or p.R1441H showed isolated nigral degeneration in the absence of LB pathology (92, 95, 96). Different domain mutations may induce different pathologies.

Neuroimaging of patients with *LRRK2* variants shows heterogeneous results. Three of the six patients with p.G2019S show a reduced heart-to-mediastinum ratio of MIBG myocardial scintigraphy (97), whereas patients with p.R1441G/H show no reduction of heart-to-mediastinum ratio (90, 92). The brain MRI commonly show no atrophic changes even over 10 years from disease onset (90, 92).

Rab GTPase, a branch of the Ras superfamily, is a crucial regulator of membrane trafficking (98). A subset of Rab proteins, including Rab3, Rab8, Rab10, and Rab12, have been reported as physiological substrates of LRRK2 (99–101). Although most pathogenic mutants of LRRK2 appear to have enhanced kinase activity toward substrates, mutations in each domain could determine the clinical phenotype and produce differential effects in terms of neuropathology. p.R1441H/G/C localized in the Rab-like ROC domain, which stimulates the LRRK2 kinase, is thought to function as a molecular switch of LRRK2 (102). The ROC domain mutant, p.R1441G, phosphorylates Rab10 more strongly than the kinase domain mutant, p.G2019S, and appears to be a potent activator of these Rab proteins (103). LRRK2 has been reported to be involved in various organelle functions and membrane dynamics in cells (104). These include mitochondria, endo-lysosomes, trans-Golgi network, microtubules, phagocytosis, endocytosis, and exocytosis of synaptic vesicles (105–112). At present, these reports do not provide a unified understanding of the molecular function of LRRK2, and the critical molecular function involved in the pathogenesis is expected to be analyzed in the future.

### *VPS13C* Gene

The *VPS13C* gene belongs to the VPS13 family, consisting of *VPS13A, VPS13B, VPS13C*, and *VPS13D* (113). The size of each gene is considerably huge, including over 70–80 exons and 200–800 kb of genomic DNA sequence (113). The *VPS13* gene is conserved from yeasts and is evolutionarily divided into four types in human. Lesage et al. (114) identified a truncated variant in *VPS13C* from a large Turkish pedigree of PD *via* linkage mapping and whole-exome sequencing (114). Patients exhibited early- or middle-age onset of PD and severe cognitive decline, with their brain pathology showing abundant expression of LB pathology. The burden analysis proved the statistical significance of variants in *VPS13C* among the Chinese early-onset PD cohorts (115). Another meta-analysis report proved the statistical significance of *VPS13C* among the Han Chinese population (116). Conversely, there is no association between *VPS13C* variants and late-onset PD (117). These findings strongly suggested that the *VPS13C* variants possibly relate to the early-onset PD and not late-onset.

The *VPS13A* variants are associated with chorea-acanthocytosis of hyperkinetic involuntary movements and abnormal morphology of erythrocytes (118). *VPS13B* variants with Cohen disease of developmental delay, microcephaly, retinal dystrophy, and intermittent neutropenia (119). *VPS13D* variants induce heterogeneous neurodegenerative disorders such as ataxia, developmental delay, spastic paraplegia, or spinocerebellar ataxia (120, 121).

It has been reported that the loss of *VPS13C* causes oxidative stress-mediated mitochondrial deterioration and upregulated PINK1/PRKN-dependent mitophagy (114). VPA13A and VPS13C are related to lipid transport between the endoplasmic reticulum and other organelles (122). VPA13A is also involved in the actin dynamics (123) and loss of VPA13A impaired autophagy and phagocytosis (124). Mitochondrial dysfunction is commonly observed in the loss-of-function of VPS13 genes and is a major pathogenic cascade to induce dopaminergic cell loss, which may be associated with the mitochondrial quality control pathway regulated by *PRKN* and *PINK1* (125, 126). Loss-of-function of *VPS13B* induces dysfunction of Golgi-trafficking (127). Loss-of-function of *VPS13D* induced peroxisome loss and mitochondrial morphological abnormality (128).

The yeast *VPS13* gene is thought to be involved in lipid transport by forming contact sites between organelles. Like yeast VPS13, the human VPS13 paralogue genes are thought to be involved in lipid transport, but the details of their molecular functions are still not clearly understood. VPS13A is associated with the endoplasmic reticulum (ER)-mitochondria contacts (122); VPS13B is mainly localized in the Golgi complex (127, 129); VPS13C is localized at ER-late endosome/lysosome contacts (122); and VPS13D is localized at ER-mitochondria and ER-peroxisome contact sites (130). They may be involved in lipid transport at the different sites, and these differences may be responsible for distinct pathophysiologies.

The neuroimaging reports of patients with *VPS13C* variants are unavailable.

### *GCH1* Gene

The *GCH1* gene was initially identified in a patient with dopa-responsive dystonia (DRD), distinctively known as Segawa's disease or DYT5a (131). The patients show unique symptoms, such as juvenile or young-age onset, dystonia initially in the feet, and excellent response to a low levodopa dosage (132). It was also reported that other symptoms include diurnal fluctuations, cramps, dystonic tremors, and sleep benefits (133). The characteristic symptoms resemble those of patients with *PRKN* or *PINK1* variants (6, 9). Patients with *PRKN* or *PINK1* also manifested the juvenile- (under 20 years of age at onset) or young-onset parkinsonism (under 40 years) with excellent response to even the low doses of levodopa, which leads to the brain pathology in the absence of LBs (6, 9).

A large population study showed a high frequency of *GCH1* variants in patients with PD compared to controls (134). The variants in *GCH1* are related to an increased risk of PD. Some GWAS also showed the association between the *GCH1* locus and PD (16, 17). In a large population study from China, *GCH1* deletions or non-coding region variants were associated with early-onset or familial PD (135). Although the *GCH1* variants are rare, they have been a proven risk factor for the onset of DRD and PD. DRD and PD may involve a common pathway causing abnormal dopamine metabolism (136).

Continuous monitoring for 32 years revealed that many patients showed no alteration or mild progression of dystonia (133), with a mild prognosis. The pedigrees primarily show autosomal dominant inheritance and female predominance (132). Some pedigrees harbor the complex appearance of patients with DRD and PD (133, 137). Adult-onset patients with *GCH1* variants show upper-limb tremors or non-tremulous parkinsonian syndrome (133). The brain pathology mostly shows the absence of LB pathology, and none to minor changes of morphological abnormalities, but only a few cases were reported (138, 139). In brief, patients with DRD and *GCH1* variants show distinctive symptoms compared to PD. The patients with PD and *GCH1* variants may involve neuronal loss in the striatum or the substantia nigra due to the reduction of dopamine transporter expression, although there are no brain pathology reports of PD phenotype with *GCH1* variants. It has been indicated in reports that “age” may be a factor in distinguishing DRD from PD. Patients with young-age onset likely belong to the DRD phenotype, whereas those with older-age onset likely belong to the PD phenotype (137). Both the disorders would be improved by oral administration of levodopa.

Studies on *GCH1* reported that half of the patients with PD show a reduction in heart-to-mediastinum ratio (137). Patients with DRD commonly showed normal values of dopamine transporter uptake in ^123^I-FP-CIT SPECT (140). However, patients with PD phenotype showed a reduction in dopamine transporter expression (134).

**Figure 1:**
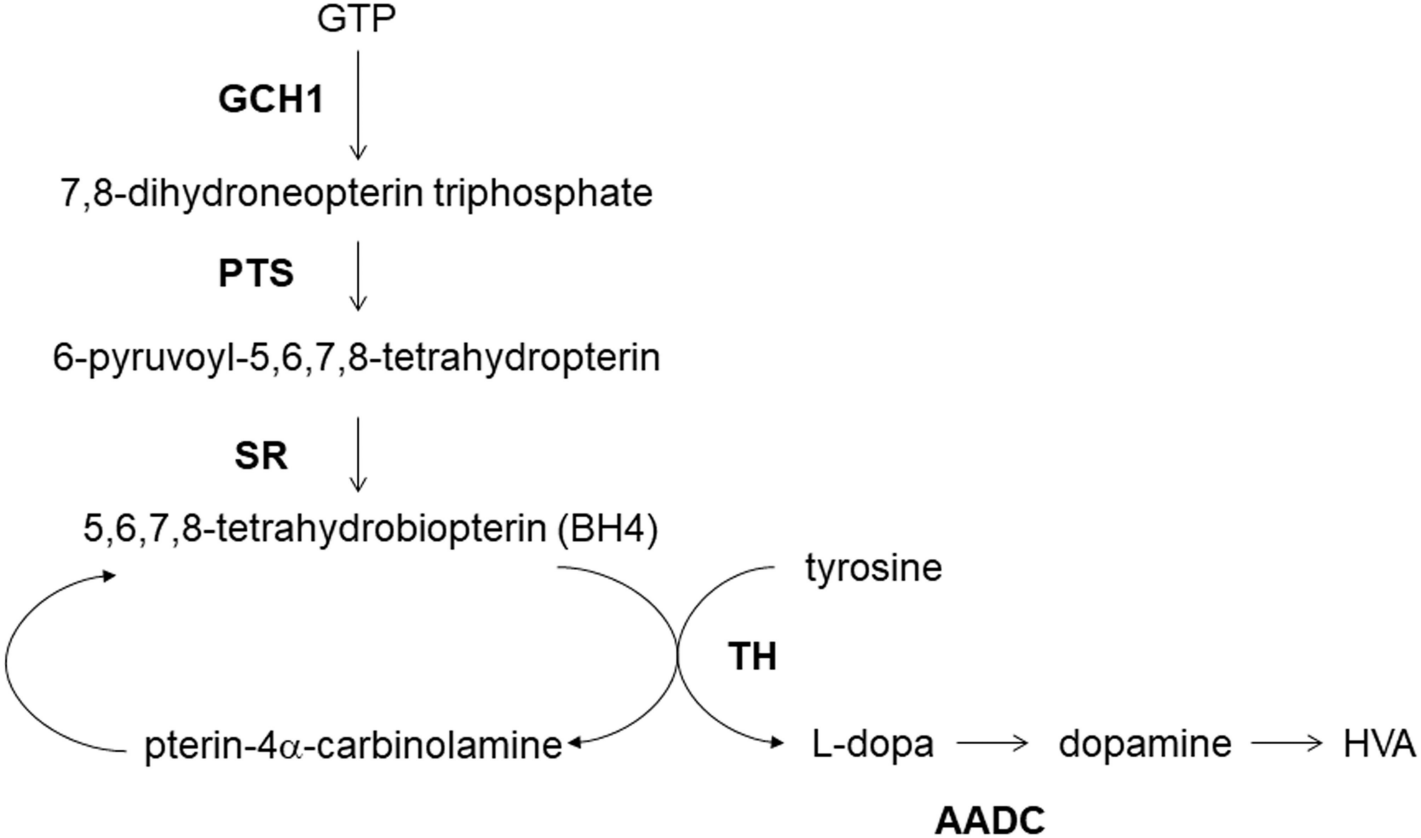
Dopamine metabolism and GCH1. GCH1, GTP cyclohydrolase 1; PTS, 6-pyruvoyltetrahydropterin synthase; SR, sepiapterin reductase; TH, tyrosine hydroxylase; AADC, aromatic L-amino acid decarboxylase; HVA, homovanillic acid.

The enzymatic deficiency of dopamine production is the main pathogenesis of DRD (141). *GCH1-*encoded GTP cyclohydrolase 1 functions upstream of the dopamine synthesis (138) (Figure 1). The deficiency of GTP cyclohydrolase 1 reduces the production of tetrahydrobiopterin, an essential co-factor in dopamine production by tyrosine hydroxylase (142). The reduction in tyrosine hydroxylase levels caused by GCH1 mutations also contributes to the symptoms related to DRD (141). Thus, deleterious variants of *GCH1* are likely responsible for the decrease in dopamine production more directly than other genes like *SNCA, LRRK2*, or *MAPT*.

### *MAPT* Gene

The *MAPT* gene, which encodes tau protein, is not a PD causative gene and is linked to frontotemporal dementia. However, *MAPT* is a gene that should not be ignored as a basis for PD pathology. Patients with *MAPT*, which was detected by GWAS, are sometimes indistinguishable from patients with PD in terms of clinical symptoms. Moreover, tauopathy is frequently observed in LRRK2 pathology, and *MAPT* variants were reported to correlate with the severity of PD (143, 144). Historically, the region of chromosome 17q21–22 has been identified as a locus related to familial frontotemporal dementia and parkinsonism by the linkage analysis (145–148). In 1998, three missense mutations and three mutations in the 5′-splice site of exon 10 in *MAPT* were identified in large Dutch kindred with hereditary frontotemporal dementia (149). Tau is fundamentally associated with multiple neurodegenerative disorders, such as Alzheimer's disease, progressive supranuclear palsy, corticobasal degeneration, frontotemporal dementia, and prion disease (150).

Patients with *MAPT* mutations showed middle-aged onset of progressive parkinsonism and cognitive decline with a high penetrance ratio (151–153). Patients likely involve psychiatric symptoms and rigid–akinesic parkinsonism (154) and show a partial response to levodopa at early-onset PD (153, 155).

Tau maintains the stability of microtubules in neurons and promotes axonal outgrowth (156). The brain pathology of patients with *MAPT* mutations shows hyperphosphorylated tau inclusions, such as neurofibrillary tangles.

It has been highlighted that patients with *MAPT* mutations or tauopathy-related disorders show no abnormalities of MIBG myocardial scintigraphy. Patients with *MAPT* mutations commonly show atrophic changes in the frontotemporal lobes in the brain MRI within a few years from disease onset. ^123^I-FP-CIT SPECT showed a severe reduction in dopamine transporter from an early stage (153, 157). Thus, patients with *MAPT* mutations may be diagnosed with PD and treated with levodopa at an early clinical stage. Our research has identified patients with *MAPT* N279K or p.K298_H299insQ from patients with middle-aged onset of parkinsonism or those clinically diagnosed with familial PD (153, 158). Tau imaging SPECT revealed a high tau accumulation from the brain stem to the basal ganglia (153). The distribution of tau pathology may relate to the onset of parkinsonism and disease severity. *In vivo*, tau imaging analysis will expand our understanding of tau-related disorders (159).

## Genetic Interactions Among Pathogenic Genes

The brain pathology of patients with *SNCA* mutations, *GBA1* variants, LRRK2 p.G2019S, or *VPS13C* variants shows LB formation. Excessive α-synuclein or α-synuclein aggregation is suggested to impair cellular vesicular transport, by which the transport of newly synthesized lysosomal enzyme GCase, encoded by *GBA1*, from the ER to the lysosomes may be inhibited (89). On the other hand, the perturbation of transport of GCase, involved in the metabolism of glycosphingolipids, could also lead to a reduction in lysosomal function and inhibit the lysosomal degradation of α-synuclein (89). This vicious cycle of *GBA1* variants has been proposed to be a risk factor for theLB formation. The *GBA1* pathogenic variants reportedly accumulate glucosylceramide and glucosylsphingosine, probably in lysosomes (160). These lipids could promote the aggregation of α-synuclein (161, 162). Nevertheless, the aforementioned considerations are speculative and await further experimental validation.

The LRRK2 was reported to inhibit the GCase activity *via* Rab10 phosphorylation in dopaminergic neurons differentiated from iPS cells harboring LRRK2 pathogenic mutations (162). Although the details of the inhibitory mechanism of GCase by Rab10 remain unknown, the reduction of the GCase activity by LRRK2 may be indirectly involved in α-synuclein accumulation and aggregation. As mentioned above, the relationship between LRRK2 and α-synuclein aggregation is complex because LRRK2 causes various pathologies, such as LB pathology, tau pathology, and TDP-43 pathology. According to a recent systematic pathological analysis, α-synuclein pathology is observed in 63.6% of *LRRK2* mutation carriers (144). On the other hand, tau pathology is found in ~100% of carriers. Most LRRK2 mutation carriers show comorbid AD pathology with amyloid-β. These observations suggest that the pathology caused by LRRK2 mutations is fundamental to neurodegenerative diseases. An interesting observation is the high frequency of AD-type phosphorylated tau accumulation (144). LRRK2 surrounds microtubules and inhibits neuronal axonal transport (110, 112). Microtubule modification by LRRK2 may affect the binding of tau to microtubules or tau phosphorylation after dissociation (163–165).

The molecular relationship between VPS13C and α-synuclein has not been elucidated so far. Because VPS13C is also localized to the lysosomes, its variant may impair lysosomal function, leading to the consequent accumulation of α-synuclein (166). Alternatively, altered lipid transport and metabolism caused by mutations in VPS13C may lead to the aggregation of α-synuclein. These possibilities should be explored in the future. Since GCH1 is involved in dopamine synthesis, it is different from the pathologies caused by the genes mentioned above. However, a report shows decreased BH4 contents in the cerebrospinal fluids of patients with LRRK2 p.N1437H and p.G2019S, and patients with sporadic PD (136). This may result from dopaminergic neurodegeneration, but it may also be possible that pathogenic LRRK2 impairs the function of GCH1.

## Perspectives

**Figure 2:**
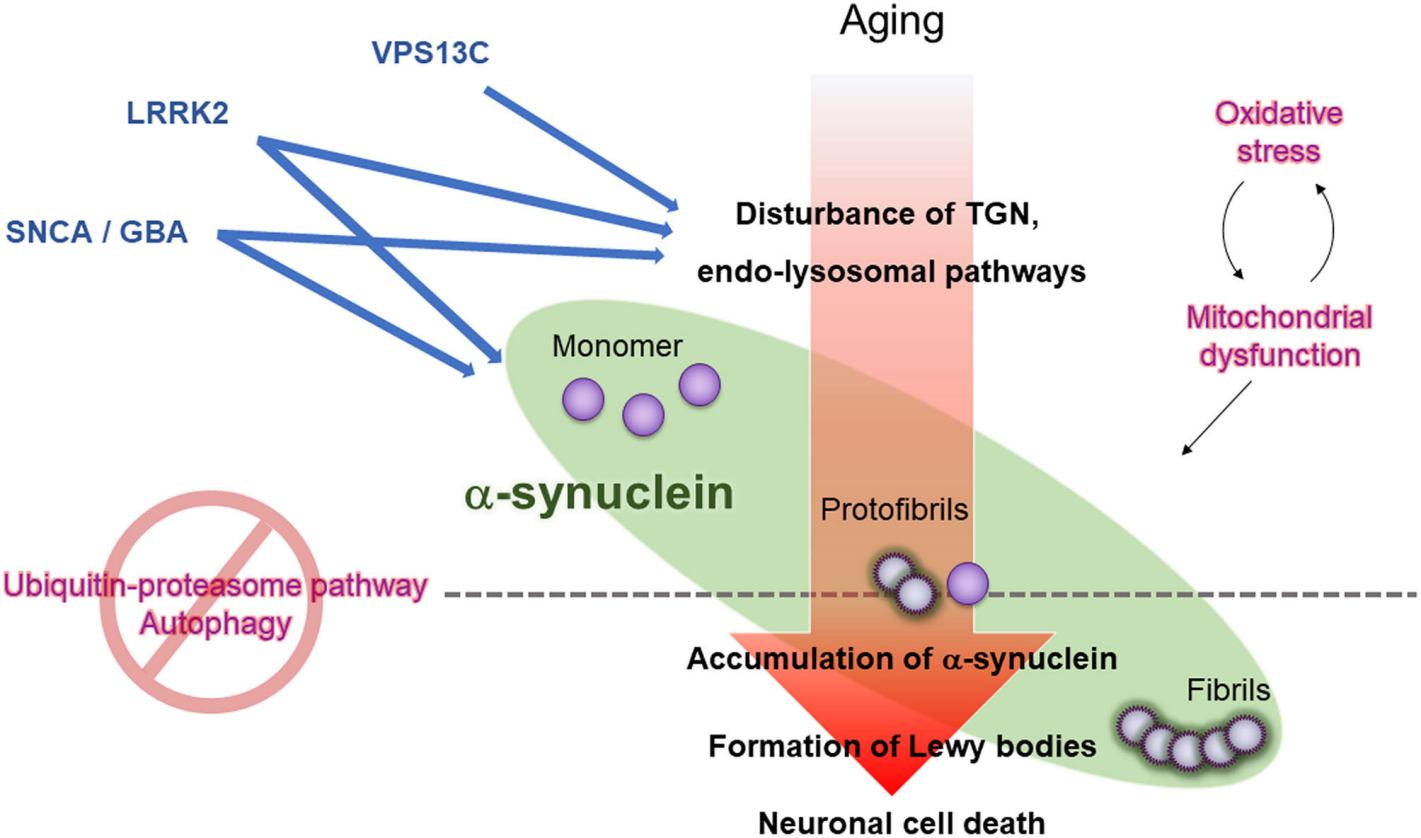
Working hypothesis for LB formation and neuronal cell death in PD. Aging, oxidative stress, and mitochondrial dysfunction lead to dysregulation of the trans-Golgi network (TGN) and endo-lysosomes in neurons, ultimately impairing the removal of the precursor of pathogenic α-synuclein (protofibrils) through ubiquitin-proteasome pathway and autophagy, promoting LB formation, and propagating pathogenic α-synuclein to neighboring neurons. Mutations in PD causative genes and risk-related genes (*VPS13C, LRRK2, SNCA*, and *GBA1*) accelerate oxidative stress, mitochondrial dysfunction, and dysregulation of the TGN and endo-lysosomes.

The GWAS has bridged the gap between molecular-based studies of familial PD and sporadic PD. The multiple genes discovered from the familial PD studies induce dopaminergic neuronal loss and the formation of LB pathology or nigral degeneration (Figure 2). The pathogenic genes yield symptoms related to parkinsonism. Moreover, “aging” is the most critical factor for the deterioration of mitochondrial maintenance or disturbance of intracellular transports during neuronal activity. However, there have been numerous unsolved questions regarding the molecular mechanism of PD pathogenesis, such as how multiple genes interact with each other to induce the dopaminergic neuronal loss, how they yield a single phenotype, what is the precise molecular model of sporadic PD, or how the genes cause LB pathology.

The next generation of GWAS research will lead to analyzing the interaction among multiple PD risk genes. As a leading example, a GWAS for the LRRK2 modifier genes has found that the WD40 protein CORO1C or DNM3 may modulate the penetrance or age-of-onset of *LRRK2* mutations (167, 168). New advances in GWASs may come from other fields of research. The loss-of-function of a preferred promoter has been reported to release its partner enhancer, which loops to a neighboring alternative promoter and activates it (169). This target switching process has been termed “enhancer release and retargeting” (169). This study shows that SNPs on the promoter of *PARK16* alter the balance of expression intensity of the genes, NUCKS1 and RAB7L1, in *PARK16* (169). This phenomenon may explain the unresolved questions about *PARK16-*mediated disease susceptibility. Thus, new concepts in genomic research can lead to novel interpretations of the data from GWAS for PD that remain mainly unexplored. On the other hand, it is challenging to identify recessively inherited PD genes such as *PRKN* and *PINK1*, which GWAS did not detect, and it is desirable to develop new methods.

A more thorough identification of risk-associated genes that cause PD will provide a clearer picture of the molecular pathogenesis of PD, yielding better and more sophisticated molecular-targeted therapies. These would include oligonucleotide therapeutics (170), antibody therapies against α-synuclein and tau (171, 172), or replacement therapies of induced pluripotent stem cells (173). Hence, a growing body of literature hints at increasing expectations for future GWAS research to help overcome PD.

## Author Contributions

KN and YI: designed the study, wrote the first draft of the manuscript, and revised the manuscript. HY, YL, MF, and NH: revised the manuscript. All authors contributed to the article and approved the submitted version.

## Funding

KN was supported by Japan Society for the Promotion of Science (JSPS KAKENHI) Grant Number 20K07893. The study was partly supported by a research grant from Biogen Japan Ltd (KN). The funders were not involved in the study design, collection, analysis, interpretation of data, the writing of this article or the decision to submit it for publication.

## Conflict of Interest

The authors declare that the research was conducted in the absence of any commercial or financial relationships that could be construed as a potential conflict of interest.

## Publisher's Note

All claims expressed in this article are solely those of the authors and do not necessarily represent those of their affiliated organizations, or those of the publisher, the editors and the reviewers. Any product that may be evaluated in this article, or claim that may be made by its manufacturer, is not guaranteed or endorsed by the publisher.
